# Elevated Atmospheric CO_2_ Modifies Mostly the Metabolic Active Rhizosphere Soil Microbiome in the Giessen FACE Experiment

**DOI:** 10.1007/s00248-021-01791-y

**Published:** 2021-06-19

**Authors:** David Rosado-Porto, Stefan Ratering, Massimiliano Cardinale, Corinna Maisinger, Gerald Moser, Marianna Deppe, Christoph Müller, Sylvia Schnell

**Affiliations:** 1 Institute of Applied Microbiology, Justus Liebig University, Giessen, DE Germany; 2 Faculty of Basic and Biomedical Sciences, Simón Bolívar University, Barranquilla, Colombia; 3 Department of Biological and Environmental Sciences and Technologies, University of Salento, Via Prov.le Monteroni, 73100 Lecce, Italy; 4 Institute of Plant Ecology, Justus Liebig University, Giessen, DE Germany; 5 School of Biology and Environmental Science and Earth Institute, University College Dublin, Belfield, Dublin, Ireland

**Keywords:** RNA metabarcoding, Elevated CO_2_, Rhizosphere microbiome, Grassland

## Abstract

Elevated levels of atmospheric CO_2_ lead to the increase of plant photosynthetic rates, carbon inputs into soil and root exudation. In this work, the effects of rising atmospheric CO_2_ levels on the metabolic active soil microbiome have been investigated at the Giessen free-air CO_2_ enrichment (Gi-FACE) experiment on a permanent grassland site near Giessen, Germany. The aim was to assess the effects of increased C supply into the soil, due to elevated CO_2_, on the active soil microbiome composition. RNA extraction and 16S rRNA (cDNA) metabarcoding sequencing were performed from bulk and rhizosphere soils, and the obtained data were processed for a compositional data analysis calculating diversity indices and differential abundance analyses. The structure of the metabolic active microbiome in the rhizospheric soil showed a clear separation between elevated and ambient CO_2_ (p = 0.002); increased atmospheric CO_2_ concentration exerted a significant influence on the microbiomes differentiation (p = 0.01). In contrast, elevated CO_2_ had no major influence on the structure of the bulk soil microbiome (p = 0.097). Differential abundance results demonstrated that 42 bacterial genera were stimulated under elevated CO_2_. The RNA-based metabarcoding approach used in this research showed that the ongoing atmospheric CO_2_ increase of climate change will significantly shift the microbiome structure in the rhizosphere.

**Supplementary Information:**

The online version contains supplementary material available at 10.1007/s00248-021-01791-y.

## Introduction

The rise of atmospheric carbon dioxide (CO_2_) concentrations and global warming are well-documented processes. Total annual anthropogenic greenhouse gas emissions have continued to increase, comprising CO_2_, which represents around 75% of these emissions [1]. Elevated CO_2_ (eCO_2_) concentrations have several consequences on plants, such as increased growth in C3, C4, and CAM plants by 41%, 22%, and 15%, respectively [2, 3]; increased plant yield [4]; decreased evapotranspiration of both C3 [5] and C4 plants [6]; augmented photosynthetic capacity [3, 7, 8]; and increased below-ground biomass [9].

Considering that nearly up to 21% of all photosynthetically fixed carbon is transferred to the rhizosphere, roots and root exudates influence the composition and biomass of soil microbiome [10, 11]. Elevated atmospheric CO_2_ increases efflux amounts of total soluble sugars, amino acids, phenolic acids, and organic acids in the root exudates [12–14]. Similarly, the rates of organic carbon as energy sources enhance microbial degradation of soil organic matter (SOC), also known as priming effect [14]. Priming effect is defined as an accelerated decomposition of SOC due to an increased supply of labile C to the soil and changes in the microbial activity as a response [15]. The microbial succession is accompanied by the activation of various, previously dormant microorganisms that respond specifically to the added substrate [15, 16].

The effects of eCO_2_ levels on soil ecosystems have been studied in free-air CO_2_ enrichment (FACE) experiments, revealing significant effects of rising CO_2_ on soil organisms. However, with regard to microbial composition and function related to carbon and nitrogen cycling, mixed results have been obtained. At the BioCON field experiment, it was found that the structure of microbial communities was different between ambient CO_2_ (aCO_2_) and eCO_2_ [17]. Likewise, the abundance of genes involved in labile C degradation and C and N fixation, as RuBisCo, carbon monoxide dehydrogenase (CODH), propionyl-CoA/acetyl-CoA carboxylase (PCC/ACC), *nifH* and *nirS* genes were significantly increased under eCO_2_ [18]. Similarly, He et al. [19] and Xiong et al. [20] have reported a shift of soil microbial communities under eCO_2_ in a soybean and a maize agro-ecosystem, respectively. These changes included stimulation of key functional genes involved in carbon fixation and degradation, nitrogen fixation, denitrification, methane metabolism, and phosphorus cycling.

Oppositely, some FACE experiments have shown no effects of eCO_2_ on soil microbiome structure and activity, as Marhan et al. [21] who described that abundances of both total 16S rRNA genes and nitrate-reducing bacteria were not influenced by CO_2_ but by sampling date and depth. Dunbar et al. [22] described that neither bacterial nor fungal community structure nor composition were altered under eCO_2_. Pujol Pereira et al. [23] did not find any significant effects of eCO_2_ on bacterial abundance, soil C, and N concentrations. Butterly et al. [24] reported that changes in microbial community structure were not detected, although eCO_2_ reduced the abundance of C and N functional genes.

The Giessen free-air CO_2_ enrichment (Gi-FACE) experiment in Giessen, Germany, has been running since 1998. It is becoming a good predictor model to assess the effects of long-term increased CO_2_ concentrations on soil microbiome structure and function. Some studies carried out in this facility aimed to assess these changes. Regan et al. [25] reported that in the Gi-FACE extractable organic carbon, dissolved organic nitrogen, NH_4_^+^, NO_3_^−^, and abundances of genes involved in ammonia oxidation and denitrification depended more on soil depth and moisture gradient than on eCO_2_. Similarly, also de Menezes et al. [26] described that increases in atmospheric CO_2_ may cause only minor changes in Gi-FACE’s soil bacterial community composition and that functional responses of the soil community are due to factors like soil moisture rather than CO_2_ concentration. Brenzinger et al. [27] reported that the abundance and composition of microbial communities in the topsoil under eCO_2_ presented only small differences from soil under aCO_2_ (aCO_2_), concluding that + 20% CO_2_ had little to no effect on the overall microbial community involved in N-cycling in the Gi-FACE soil. More recently, Bei et al. [28] described that eCO_2_ had significant effects on the functional expression associated to both rhizosphere microbiomes and plant roots; and that abundances of Eukarya relative to Bacteria were significantly decreased in eCO_2_ as well.

The question of why some studies reported differences between eCO_2_ and aCO_2_ while some others did not is still open. Several abiotic and biotic factors could be the reason for the contradictive observations in the different experimental setups described above. However, all such previous studies conducted in the Gi-FACE used a DNA-based metagenomic approach, with the exception of Bei et al. [28], who utilized a metatranscriptomic approach. The disadvantage of using DNA is that, after a cell dies, amplifiable extracellular DNA can remain in soils for weeks to years and may bias DNA-based estimates of the diversity and structure of soil microbial communities [29, 30]. Moreover, Carini et al. [31] reported that DNA from dead cells or free DNA represented a large fraction of microbial DNA in many soils, comprising approximately 40.7% and 40.5% of amplifiable prokaryotic 16S rRNA genes and fungal ITS amplicons, respectively. Therefore, DNA-depending studies may overestimate the richness of the soil microbiome by up to 55% for prokaryotes and 52% for fungi [31] and in consequence may hide the active microorganisms that are involved in soil microbial processes.

A better approach for assessing differences between eCO_2_ and aCO_2_ is the use of RNA instead of DNA for 16S rRNA metabarcoding analysis. The ribosome numbers are correlated to the metabolic activity of bacteria [32], and different studies showed that, with this approach, the active organisms instead of the dormant ones were assessed [33–35]. Additionally, results of the metatranscriptomic methodological approach on the Gi-FACE soil microbiome reported by Bei et al. [28] demonstrated that RNA instead of DNA is a better predictor of microbiome composition and activity. For this reason, the aims of the present work were (i) to evaluate the effect of long-term eCO_2_ concentrations and increased C supply on active soil microbiome through an rRNA-based metabarcoding approach; (ii) to assess the differences between eCO_2_ and aCO_2_ conditions in rhizosphere and bulk soils; and (iii) to link these differences with environmental factors.

The following questions have been addressed:

1. Is the community structure of active bacteria different between ambient and elevated CO_2_ in rhizosphere and/or bulk soil?
2. Which other environmental parameters beside CO_2_ shape the community?

## Material and Methods

### Study Site Description

The Gi-FACE study is located at 50° 32′ N and 8° 41.3′ E near Giessen, Germany, at an elevation of 172 m above sea level. It consists of three pairs of rings with a diameter of 8 m; each pair consists of an ambient and an elevated CO_2_ treatment ring [36]. Since May 1998 until present, elevated CO_2_ rings have been continuously enriched by 20% above ambient CO_2_ concentrations during daylight hours. Ambient and elevated CO_2_ rings are separated by at least 20 m, and each pair is placed at the vertices of an equilateral triangle. The presence of a slight slope within the experimental site (between 0.5 and 3.5°) places the rings on a moisture gradient, such that pair 1 has the lowest mean moisture content (38.8% ± 10.2%) and pair 2 has the highest mean moisture content (46.1% ± 13.2%), whereas pair 3 is intermediate (40.7% ± 11%) [26, 36]. The average annual air temperature and precipitation are 9.4 °C and 580 mm, respectively.

The vegetation is an *Arrhenatheretum elatioris* Br.Bl. *Filipendula ulmaria* subcommunity, dominated by *Arrhenatherum elatius, Galium album*, and *Geranium pratense*. At least 12 grass species, 15 non-leguminous herbs and up to 5 legumes with small biomass contributions (< 5%) are present within a single plot [37]. The experimental field has not been ploughed for more than 100 years. It has received N fertilization in form of granular mineral calcium ammonium nitrate (40 kg N ha^−1^ year^−1^) once a year since 1995 and has been mown twice a year since 1993. The soil at the Gi-FACE site is classified as Fluvic Geysol; its texture is a sandy clay loam over a clay layer, with pH = 6.2 and average C and N contents of 4.5% and 0.45%, respectively, as measured in 2001 [36].

### Soil Sampling and Physico-chemical Parameter Measurements

Soil sampling was performed utilizing sawed off 50 ml syringes (11 × 3 cm), and four samples were taken to a depth of ~ 10 cm within each ring in September 2015. Soil cores were gently shaken by hand to remove loosely attached soil (bulk soil), while the soil that remained attached to the roots was considered as rhizosphere soil. Bulk and rhizosphere soils were sieved (< 2 mm) and stored at − 80 °C for further analyses. Samples from each soil core were classified in four groups considering the CO_2_ conditions (ambient and elevated) and the soil habitat (bulk soil and rhizosphere soil).

Ammonium and nitrate concentrations were measured according to Kandeler et al. [38] and Bak et al. [39]. Water content, dry matter, and water holding capacity of soil samples were measured gravimetrically [40]. Carbon and nitrogen content of soil were measured by pyrolysis coupled to gas chromatography on a EA 1100 elemental analyzer (ThermoQuest, Milan, Italy) using a TCD detector by the Dumas method according to HBU (1996) [41] and VDLUFA (2012) method [42]. Injected CO_2_ and CO_2_ soil fluxes were determined from August to September 2015. Injected CO_2_ was measured at 60 cm above ground with an infrared gas analyzer (LI-COR 6252) [36]. CO_2_ soil fluxes were measured weekly using an automated closed dynamic chamber system (LI-COR 8100, LI-COR Inc., Lincoln, Nebraska, USA). Per ring, 4 PVC soil collars (20.3 cm diameter) were permanently installed as chamber bases in 2006 and held vegetation free since 2008. Fluxes were calculated from the increase in CO_2_ concentration in the chamber over the 1–3 min closure time as described by Keidel et al. [43].

Central tendency and dispersion measures were calculated for soil chemical data. CO_2_ injection and CO_2_ fluxes data were analyzed using growth curve analysis (GCA) [44], with R packages gazer version 0.1 [45] and lme4 version 1.1–23 [46], creating polynomial-transformed predictor variables, fitting them to a linear mixed model by maximum likelihood, and assessing differences between CO_2_ conditions with a t-test, using an alpha of < 0.05.

### RNA Extraction and Reverse Transcription

RNA extraction was performed following a modified protocol of Mettel et al. [47]. For the extraction, 0.3–0.5 g of soil were weighed in reaction tubes containing 100 mg of sterile zirconia beads, added with 700 µl TPM buffer (50 mM Tris–HCl (pH 5), 1.7% [wt/vol] polyvinylpyrrolidone, 20 mM MgCl_2_), and vortexed for 30 s. Cells were then disrupted in a cell mill MM200 (Retsch, Haan, Germany) for 2 min at a frequency of 30 Hz. Soil and cell debris were precipitated by centrifugation in a microcentrifuge (Heraeus Fresco, Thermo Fisher Scientific Inc., Waltham) for 5 min at 17,000 g and 4 °C, and then the supernatant was transferred into a fresh reaction tube. To the resulting soil pellet 700 µL of buffer PBL (5 mM Tris–HCl (pH 5), 5 mM Na_2_EDTA, and 0.1% [wt/vol] sodium dodecyl sulfate) were added, and the disruption process was performed again as described above. Both supernatants from the lysis processes were pooled in one reaction tube.

The pooled supernatant was immediately extracted, initially with the addition of 500 µl of phenol/chloroform/isoamyl alcohol (25:24:1) and subsequent with chloroform/isoamyl alcohol (24:1). Afterwards, each time the sample was centrifuged for 5 min at 17,000 g and 4 °C. The resulting upper aqueous phase was transferred to a new reaction tube, 800 µl of PEG solution was added (30% [wt/vol] polyethylene glycol 6000 and 1.6 M NaCl), incubated in ice for 30 min and centrifuged for 30 min at 17,000 g and 4 °C. Subsequently, the DNA/RNA pellet was washed with 800 µl of ice-cold 75% ethanol, dried out and dissolved in 50 µl of nuclease free water.

After extraction, samples were treated for DNA digestion with RNase-Free DNase Set (QIAGEN GmbH — Germany) according to manufacturer instructions; DNase reaction was stopped with 10 µl of 50 mM EDTA. With the DNA-free RNA, a PCR was carried out, using the universal 16S rRNA gene primers 27F (5’-AGAGTTTGATCMTGGATCMTGGCTCAG-3’) and 1492R (5’- GGTTACCTTGTTACGACTT-3’) [48, 49] and checked on agarose gel electrophoresis to verify the absence of remaining DNA in the samples. Subsequently, reverse transcription was performed utilizing AccuScript High Fidelity 1st Strand cDNA Synthesis Kit (Agilent Technologies, Inc., Cedar Creek, TX, USA) following manufacturer instructions.

### 16S rRNA Ion Torren Sequencing and Metabarcoding Analysis

The 16S rRNA gene hypervariable regions (V4&V5) were PCR amplified using the set of primers 520F (5’-AYTGGGYDTAAAGNG-3’) [50] and 907R (5’-CCGTCAATTCMTTTRAGTTT-3’) [51] and sequenced by Ion Torrent technique following the protocol described by Kaplan et al. [52]. The obtained Ion Torrent sequencing output was analyzed using QIIME2 version 2020.6.0 [53], sequences were demultiplexed with the QIIME2 cutadapt command [54] using a barcode error rate of 0 and assigned to specific samples by corresponding barcodes. Later, quality control, denoising, sequences dereplication, and chimera filtering were performed using DADA2 software [55]; the first 15 nucleotides were trimmed, and sequences were truncated at a position of 320 nucleotides. Amplicon sequence variants (ASV) generated with DADA2 were taxonomically affiliated with a trained fitted classifier [56, 57] based on the SILVA 138 database [58, 59].

Alpha and beta diversity analyses were performed using R studio software 1.1.419, R packages Phyloseq 1.22.3 [60] and Vegan 2.4–6 [61]. Before diversity analyses, ASVs were collapsed by genera. For alpha diversity assessment, rarefaction was applied and diversity indices (Observed species, Simpson, Shannon, Fisher) were calculated and compared among CO_2_ conditions and soil habitats using the Wilcoxon test [62] with the Bonferroni correction method through 999 permutations. For non-constrained beta diversity analyses, data were transformed using centered log ratio (clr) method [63, 64], using R package Aldex2 1.18.0 [65]. Later, community dissimilarity distance matrices were created using the Aitchison distance [63, 64] and visualized using principal component analysis (PCA) [66]. Statistical differences among treatments, rings, and CO_2_ conditions were assessed by a permutational multivariate analysis of variance using Adonis method and employing 999 permutations [67]. Additionally, the degree of dispersion of the bacterial community composition from the four soil cores taken in each ring was assessed as described above. Redundancy analysis (RDA) was used to explore associations between microbial community structures and environmental parameters, and a permutation test of redundancy analysis using 999 permutations was applied for evaluating their statistical significance [68].

For the analysis of correlation between bacterial genera and environmental parameters, the genera belonging to the core microbiome of each of the soil sample groups were calculated, and their counts were transformed to relative abundance with package Microbiome version 1.8.0 [69]. Later, core microbiomes were calculated including genera with a total relative abundance of ≥ 0.01% and present in ≥ 85% of the corresponding group’s samples. A correlation test was performed using Aldex2 1.18.0 [65] and its “aldex.corr” function, utilizing Pearson's correlation coefficient, and p values were corrected using false discovery rate (FDR) method with an alpha of < 0.05.

Differential abundance of genera from rhizosphere soils was assessed by comparing the core microbiomes of each CO_2_ condition utilizing the R packages DESeq2 1.24.0 [70] and Aldex2 1.18.0 [65]. DESeq2 analysis was performed by estimating the size factor and the dispersion using the geometric mean of the core microbiome genera; later, values were fitted with a generalized linear model using negative binomial distribution and applying a Wald significance tests, the option “local” for fitting of dispersions to the mean intensity and an alpha threshold of < 0.05. Aldex2 analysis was done by performing a centered log ratio (clr) transformation using as denominator the geometric mean abundance of all features and 128 Monte Carlo instances; later, a Welch’s t-test with a Benjamini–Hochberg correction and threshold < 0.05 was performed.

Functional capabilities based on the obtained 16S rRNA data were predicted using PICRUSt2 version (v2.3.0 beta) [71]. PICRUSt2 analysis was carried out using the default pipeline option. Afterward, EC number, KO functions, and MetaCyc non-constrained beta diversity and differential abundance analyses were performed as described above.

### Quantitative PCR

The quantification of 16S rRNA gene to estimate total bacterial abundance was performed following the protocol described by Kaplan et al. [52], but instead of DNA, cDNA products described above were used for the quantification. Quantitative PCR (qPCR) was conducted on a Rotor-Gene Q (Qiagen, Hilden, Germany) by using Absolute qPCR SYBR Green Mix (Thermo Fisher Scientific). Statistical comparisons were done with Kruskal–Wallis and Wilcoxon tests with the Benjamini–Hochberg adjustment method using R Package stats version 3.6.3.

## Results

### Ion Torrent Sequencing

A total of 5,855,099 raw sequences were obtained. After demultiplexing, sequences were assigned to each sample, ranging sequence counts in each sample from 306,675 to 22,410. After quality control, denoising, sequence dereplication, and chimera filtering with DADA2 software, 2,674,159 sequences were removed, resulting in 3,180,940 non-chimeric sequences and 11,587 representative sequences which were grouped into ASVs (Amplicon sequence variations) at a 99% similarity. Later, sequences belonging to chloroplast and mitochondria were removed, resulting in 11,508 ASVs.

### Soil Microbial Diversity

**Fig. 1:**
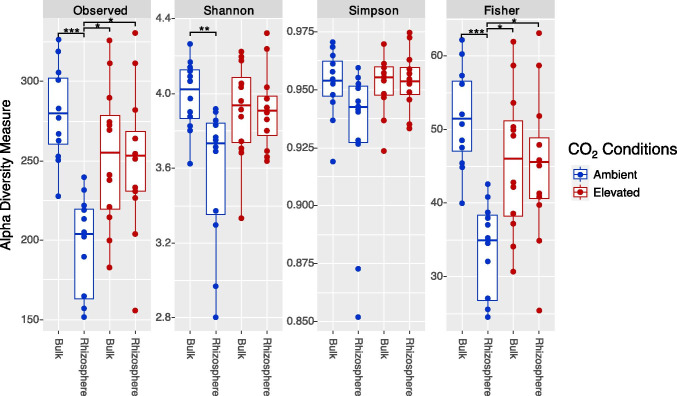
Alpha diversity metrics. aCO_2_, ambient CO_2_ conditions; eCO_2_, elevated CO_2_ conditions. * p smaller 0.01, ** p smaller 0.001, *** p smaller 0.0001

Diversity indexes were evaluated to assess differences in soil microbiome between eCO_2_ and aCO_2_ conditions. In the Gi-FACE, soil active bacterial diversity changed due to the influence of increased concentrations of CO_2_ (Fig. 1). These changes are better appreciated when comparing bulk and rhizosphere soil fractions from aCO_2_ and eCO_2_ rings separately. In regard to alpha diversity of rhizosphere and bulk soil fractions from aCO_2_ rings, significantly higher diversity values were observed in bulk compared to rhizosphere soils with Observed species (p value 0.00036), Shannon (p value 0.0086), and Fisher (p value 0.00036) indexes (Fig. 1). Nevertheless, this difference was not detected between bulk and rhizosphere soil fractions from eCO_2_ rings, indicating an evenness between the rhizosphere and bulk soils in eCO_2_ rings (Fig. 1). Likewise, eCO_2_ rhizosphere soil presented greater diversity values in comparison to its aCO_2_ counterpart, according to Observed species (p value 0.0193) and Fisher (p value 0.0193) indexes.

A distance matrix was created using the Aitchison distance and later ordinated using the principal component analysis (PCA) to further analyze the microbiome composition. Initially, the dispersion of the four soil cores taken within each ring and their distance to the centroids was assessed. They indicated a considerably different soil microbiome composition in each soil core, even when soil cores of the same rings were compared (S1). On the other hand, the assessment of differences among the evaluated habitats showed that the strongest effect on the bacterial microbiome differentiation in the soil was the ring factor, either for rhizosphere or bulk soils (p value 0.001).

**Fig. 2:**
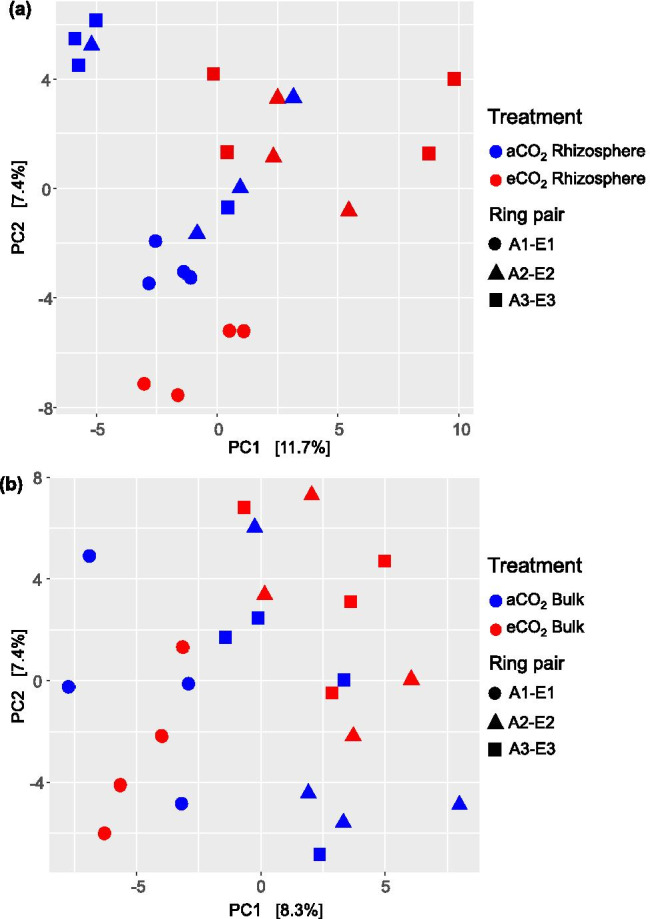
Principal component analysis (PCA) calculated based on Aitchison community dissimilarity distance matrix of **a** rhizosphere soils from ambient and elevated CO_2_ rings and **b** bulk soils from ambient and elevated CO_2_ rings. A, ambient CO_2_ rings; E, elevated CO_2_ rings; aCO_2_, ambient CO_2_ conditions; eCO_2_, elevated CO_2_ conditions

Similarly, there were significant differences among the community composition of the four evaluated groups (aCO_2_ bulk soil, aCO_2_ rhizosphere soil, eCO_2_ bulk soil, eCO_2_ rhizosphere soil) (p value 0.001). In the same way, the PCA showed a clear differentiation between the microbiome composition of the rhizospheres from eCO_2_ and aCO_2_ rings (p value 0.002) (Fig. 2a). On the contrary, the separation of the microbial community composition between the bulk soils from aCO_2_ and eCO_2_ rings was not clear and statistically not significant (p value 0.327) (Fig. 2b).

### Effect of Environmental Parameters on Microbial Community

**Fig. 3:**
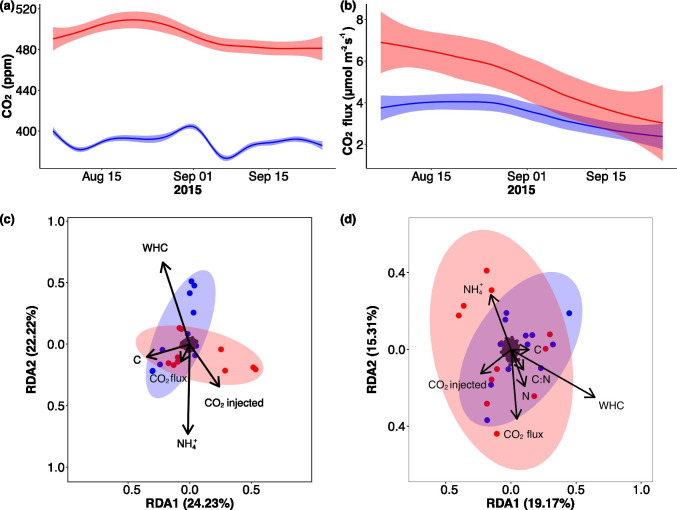
Time series data from August to September 2015 of **a** average environmental CO_2_ concentrations and **b** average soil CO_2_ fluxes of ambient (blue) and elevated (red) CO_2_ conditions; level of confidence interval of 0.95. Redundancy analysis (RDA) based on Aitchison community dissimilarity distance matrix of **c** rhizosphere soils from ambient (blue) and elevated (red) CO_2_ rings and **d** bulk soils from ambient (blue) and elevated (red) CO_2_ rings; black dots indicate soil bacterial genera

A redundancy analysis (RDA) was carried out to assess the effect of environmental factors on the soil microbiome of the Gi-FACE. The results indicated that continuously higher environmental CO_2_ concentration was a factor that exerted a significant effect on the differentiation of the microbial communities of eCO_2_ rings (p value 0.021) (Table 1, Fig. 3a). Furthermore, CO_2_ soil fluxes on average were 35% higher in eCO_2_ rings in comparison to the aCO_2_ ones, and this difference was statistically significant throughout the assessed period of time (p value 0.031) (Fig. 3b). Moreover, increased soil fluxes of CO_2_ are associated with the differences that were observed in Gi-FACE soil microbiome (p value 0.001).

**Table 1:** p values of permutation test for redundancy analysis (RDA) under reduced model using an Aitchison community dissimilarity distance matrix

Environmental parameter	Whole soil	Rhizosphere soil	Bulk soil
C:N	0.025 *	––-	0.127
CO_2_ injected concentration	0.021 *	0.010 **	0.097
CO_2_ flux concentration	0.001 ***	0.003 **	0.004 **
NH_4_^+^	0.001 ***	0.002 **	0.018 *
Total carbon	0.001 ***	0.007 **	0.001 ***
Water holding capacity	0.006 **	0.024 *	0.100
Total nitrogen	0.141	––-	0.688

Significance codes: 0.0001 ‘***’, 0.001 ‘**’, 0.01 ‘*’

Likewise, the ammonium content in the whole soil, rhizosphere and bulk soil fractions had a significant influence on the community composition (Table 1), despite the fact that soil ammonium concentrations were not significantly different between eCO_2_ and aCO_2_ rings (p value 0.313) (S2). Similarly, the total carbon content had significant influence on the whole soil and bulk soil microbial community structure (Table 1), but likewise ammonium there were no significant differences in carbon content between aCO_2_ and eCO_2_ rings (p value 0.1304) (S2). On the contrary, the average carbon/nitrogen ratio in the whole soil of the eCO_2_ rings (11.1:1) was significantly higher in comparison with aCO_2_ rings (10.69:1) (p value 0.0069) and had a significant effect (p value 0.025) on the microbial community composition (Table 1).

Furthermore, when observing each habitat separately, the RDA indicated that in the rhizosphere soil, the CO_2_ atmospheric concentration had a significant effect on the microbiome differentiation between the aCO_2_ and eCO_2_ rings (p value 0.010) (Table 1, Fig. 3c). In contrast, eCO_2_ had no substantial influence on the composition of the microbial community’s structure of the bulk soils (p value 0.097) (Table 1, Fig. 3d).

Correlation analysis between environmental variables and rhizosphere soil core microbiome demonstrated that the abundance of several bacterial genera was either positively or negatively correlated with environmental CO_2_ concentrations and soil CO_2_ fluxes. Among the main bacterial families that were significantly positively correlated with environmental eCO_2_ and soil CO_2_ fluxes concentrations are Rhodanobacteraceae*,* “Labraceae,” Xanthomonadaceae, Rhodobacteraceae, Rhizobiaceae, Pseudomonadaceae, Phaselicystidaceae, Haliangiaceae, Bacillaceae, Streptomycetaceae, Xanthobacteraceae, Burkholderiaceae, Devosiaceae, Haliangiaceae, Comamonadaceae and Polyangiaceae (Table S3.1)*.* On the contrary, families “Solibacteraceae,” Caulobacteraceae, Acetobacteraceae, Thermoactinomycetaceae, Beijerinckiaceae, and Blastocatellaceae were negatively correlated with environmental eCO_2_ and soil CO_2_ fluxes (Table S3.1). Moreover, bacterial orders Nitrospirales, Caulobacterales, “Rokubacteriales,” Vicinamibacterales, “Tistrellales,” and “Rokubacteriales” were significantly correlated with NH_4_^+^ content and soil water holding capacity in rhizosphere soils (Table S3.1).

### Changes on the Rhizosphere Microbial Community Composition

**Fig. 4:**
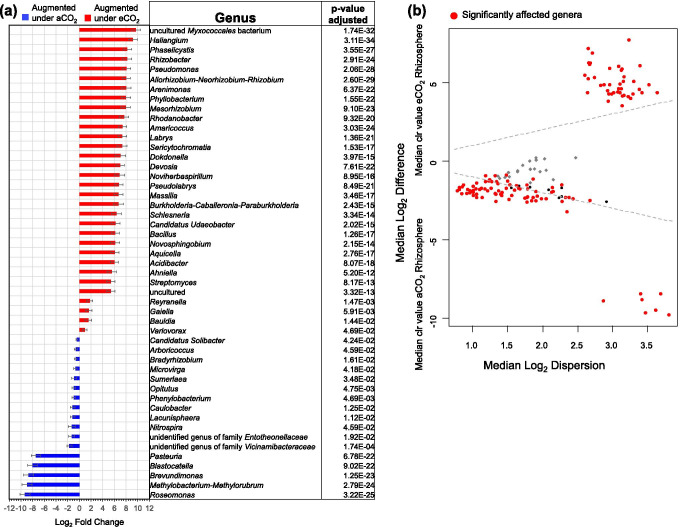
Differential abundances of core microbiome bacterial genera of rhizosphere soil under elevated and ambient CO_2_. **a** DESeq2 test results with an alpha threshold < 0.05 and error expressed as standard error of log fold change. **b** Aldex2 results using centered log ratio (clr) transformation and the geometric mean abundance of all features; red points indicate significantly different genera after Welch’s t-test and Benjamini–Hochberg correction with an alpha threshold < 0.1

Differential abundance analyses demonstrated that several rhizosphere soil genera were affected. Both Aldex2 and DESeq2 demonstrated that 42 bacterial genera were stimulated under eCO_2_, among those are *Haliangium*, *Phaselicystis*, *Rhizobacter*, *Pseudomonas*, *Rhizobium*, *Phyllobacterium*, *Mesorhizobium*, *Rhodanobacter*, *Labrys*, unidentified genus of the class *“*Sericytochromatia,” *Dokdonella*, *Massilia*, *Burkholderia*, *Bacillus*, *Novosphingobium*, *Acidibacter*, and *Streptomyces* (Fig. 4a, Fig. 4b)*.* These genera showed Log_2_ Fold changes ranging from 0.910 to 9.67*.* Furthermore, Aldex2 test showed that other 56 genera were significantly stimulated in the rhizosphere soil of eCO_2_ rings. These genera belonged mainly to bacterial families Nocardioidaceae, Beijerinckiaceae, Pyrinomonadaceae, “Koribacteraceae”, “Xiphinematobacteraceae,” Propionibacteriaceae, Dongiaceae, Geminicoccaceae, Solirubrobacteraceae, Blastocatellaceae, Caulobacteraceae, Nitrosomonadaceae, Xanthobacteraceae, Caulobacteraceae, Fibrobacteraceae, Acetobacteraceae*,* unidentified family of the phylum “Latescibacterota,” Myxococcaceae*,* “Solibacteraceae”, Rhizobiaceae, and Gemmatimonadaceae (S3).

On the contrary, both differential abundance tests indicated that some genera presented a decreased of abundance under eCO_2_ conditions. Among these are unidentified genus of the family Vicinamibacteraceae, *Pasteuria*, *Caulobacter,* unidentified genus of the family *“*Entotheonellaceae,” *Brevundimonas*, *Methylobacterium*-*Methylorubrum*, *Sumerlaea*, *Blastocatella*, *Phenylobacterium*, *Lacunisphaera*, *Roseomonas*, and *Opitutus.* These genera had Log_2_ Fold changes from − 0.421 to − 9.31 in eCO_2_ ring (Fig. 4a, Fig. 4b, S3).

### Functional Metagenomics Prediction

Beta diversity results of functional capabilities based on 16S rRNA data showed significant differences on functional metagenome’s composition of rhizosphere soils from aCO_2_ and eCO_2_ conditions. PICRUSt2 predicted functional metagenome were different regarding Enzyme Commission number (EC number) (p value 0.005), KEGG Orthology (KO) for molecular functions (p value 0.019), and MetaCyc Metabolic Pathways (p value 0.022) (S4). Moreover, similar to taxonomical results, predicted bulk soil’s functional metagenomics from aCO_2_ and eCO_2_ conditions did not show great differences regarding its beta diversity, neither on EC numbers (p value 0.197), KEGG Orthology (p value 0.179), or MetaCyc Metabolic Pathways (p value 0.317) (S4).

Besides, the analyses of significantly affected predicted enzymes indicated that several enzymes of degradation of carbon compounds were significantly stimulated in rhizospheric soils under eCO_2_ conditions. Enzymes involved in carbohydrates, lipids, amino acids, and polycyclic aromatic hydrocarbon degradation were significantly stimulated. Additionally, numerous predicted enzymes and pathways of synthesis of cellular components, membrane transporters, and quorum sensing were significantly higher under eCO_2_ conditions (S5). Also, according to KEGG Orthology for molecular functions, several enzymes involved in nitrogen fixation, nitric-oxide synthesis, and nitrite and nitrate reduction were predicted to be more abundant in eCO_2_ rhizosphere soil (S5).

### Quantitative PCR

**Fig. 5:**
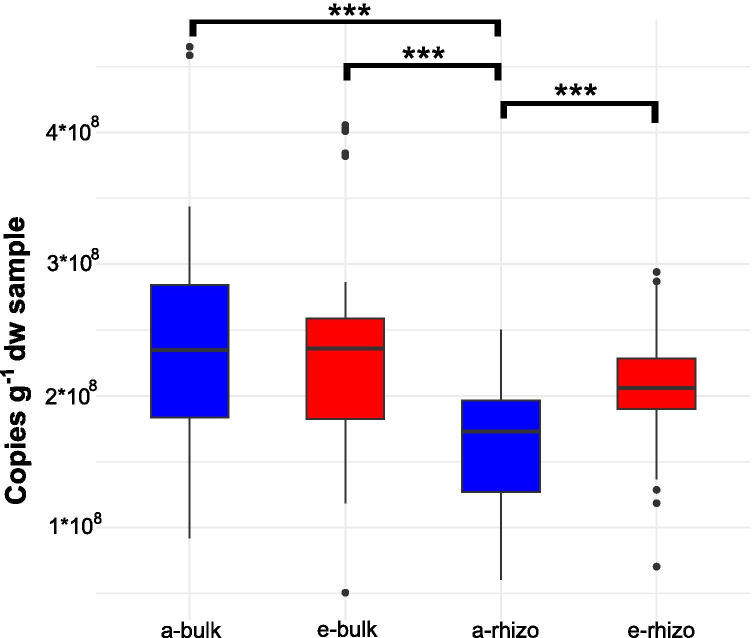
Boxplot of 16S rRNA quantification of ambient CO_2_ rings bulk soil (a-bulk), elevated CO_2_ rings bulk soil (e-bulk), ambient CO_2_ rings rhizosphere soil (a-rhizo) and elevated CO_2_ rings rhizosphere soil (e-rhizo). Significance codes: 0.0001 ‘***’

Active biomass estimation by 16S rRNA quantification demonstrated changes due to eCO_2_ concentrations. A 20% increase of 16S rRNA copy numbers per g dry weight soil in eCO_2_ rhizosphere (2.07 ± 0.50*10^8^) in comparison to aCO_2_ rhizosphere (1.66 ± 0.44*10^8^) was observed (p value 0.0001). Nevertheless, when comparing the 16S rRNA copy numbers per gram dry weight soil of bulk soils from aCO_2_ (2.35 ± 0.80*10^8^) and eCO_2_ (2.35 ± 0.79*10^8^) conditions, no significant differences were found (p value 0.9588) (Fig. 5). Moreover, significant differences were found between bulk and rhizosphere soils from aCO_2_ (p value 2.1 * 10^–5^) with in average 29% more copies per dry weigh in bulk soil compared to rhizosphere soil. Nonetheless, when comparing rhizosphere and bulk soils from eCO_2_ rings, this difference is lower and not significant (p value 0.1455), with the bulk soil having 12% more copies than the rhizosphere soil (Fig. 5).

## Discussion

### Changes in Microbiome Structure and Composition

Elevated CO_2_ concentrations affect the composition and biomass of soil microbial communities in the rhizosphere because of greater inputs of labile carbon (C) via root exudation may increase the microbial N demand. This causes an increased competition between plants and soil microorganisms for available N; therefore, N dynamics are likely to change under eCO_2_ [10, 11, 14, 72].

Our results showed that eCO_2_ had a strong effect in the Gi-FACE on the metabolic active microbiome of the rhizosphere soil, in contrast to the microbiome of the bulk soil which remained mostly unaffected. Alpha diversity indices indicate that a shift occurred under eCO_2_ conditions, producing an evenness in terms of alpha diversity between rhizosphere and bulk soil. Since significant differences were found between bulk and rhizosphere soil of aCO_2_ rings, this evenness represents an increase in alpha diversity of eCO_2_ rhizosphere soil (Fig. 1). Furthermore, beta diversity results revealed a different abundance and microbial community composition in the rhizosphere of eCO_2_ rings compared to aCO_2_ rings (Fig. 2).

These results differ from previous reports of the eCO_2_ effects on the Gi-FACE soil microbiome, which stated that only subtle or no effect occurred on microbial communities and that the differences were mostly due to soil conditions and the moisture gradient that occurs at this facility [25–27]. Similarly, to the aforementioned studies, our data confirmed that samples from ring-pair A1-E1 had lower water content in comparison to A2-E2 and A3-E3 samples (S2), and that water holding capacity (WHC) significantly influenced the soil microbiome (Table 1, Fig. 3). This was observable in the effect that the ring-pair factor had on the beta diversity of the Gi-FACE soil microbiome (p value 0.001) (Fig. 2).

Nonetheless, besides the moisture gradient, the observed differences caused by the eCO_2_ were likely detected due to the RNA-based metabarcoding approach used in our work, which is able to differentiate the metabolic active microorganisms from the inactive ones, avoiding the biases caused by DNA of dead cell or extracellular DNA, which can comprise approximately 41% of the amplifiable prokaryotic 16S rRNA genes in soil [31]. However, RNA metabarcoding has its limitations as well, mainly due to the fact that RNA conversion to cDNA requires the use of a reverse transcriptase which lacks proofreading activity, creating point mutations in some of the cDNA sequences [73]. Reverse transcriptase also regularly performs template switching, which can lead to the production of chimeric cDNA sequences and the creation of shortened isoform sequences from intramolecular template switching [74, 75]. Nevertheless, in our study these limitations were minimized by using a Moloney murine leukemia virus reverse transcriptase (MMLV-RT) derivative combined with a *E. coli* DNA polymerase III ε subunit which lowers the reverse transcription error rate by threefold, and later the resulting cDNA was amplified with a proofreading DNA polymerase which produced up to eightfold fewer errors [76].

The study of Bei et al.[28], which also addressed the active microbial community by using a metatranscriptomic approach, supports our results. For the summer of 2015, the same year that we took the samples for this study, they reported significant effects of eCO_2_ on the functional expression related to rhizosphere and plant roots associated microbiomes in the Gi-FACE. Also, similarly to our work, they described that the increase in bacterial abundance was related to significant enrichment of different taxonomical groups, including Acidobacteria, Actinobacteria, and Proteobacteria, and changes related to a significant decrease in Fungi and increase in Actinobacteria.

However, Bei et al. [28] found no significant eCO_2_ effect on the rhizosphere soil and root-associated microbiomes during the summer of 2017. These contrasting results for different years may result from climatic conditions in summer, since the summer 2015 was characterized by prolonged heat waves, while the mean temperature in summer 2017 was closer to the long-term average. The effect of eCO_2_ on the soil rhizosphere microbiome we found in our study may be affected by the above average temperatures of this particular year. Additionally, the prediction that heat waves will occur more frequently in the future [77] emphasizes the importance of our findings.

The reason why only the rhizosphere microbiome, in contrast to the bulk soil microbiome, was affected by eCO_2_ influx is most probably a consequence of the priming effect of the increased flux of roots exudates and consequently higher availability of carbon compounds. This increased supply of labile C causes an accelerated decomposition of soil organic C [15], which activates previously dormant microorganisms [15, 16].

### Effect of eCO_2_Concentration on Microbial Community, C and N Cycles

Our results of the effect of environmental parameters on soil microbiome composition demonstrated that several rhizosphere bacterial families such as Rhodanobacteraceae, “Labraceae,” Xanthomonadaceae, Rhodobacteraceae, Rhizobiaceae, Pseudomonadaceae, Phaselicystidaceae, Haliangiaceae, Bacillaceae, Streptomycetaceae, Xanthobacteraceae, Burkholderiaceae, Devosiaceae, Haliangiaceae, Comamonadaceae, and Polyangiacea were positively correlated with eCO_2_ fumigation and soil CO_2_ fluxes. Within these families are found bacterial genera as *Streptomyces, Burkholderia, Dokdonella, Bacillus, Pseudolabrys, Devosia, Mesorhizobium, Acidibacter, Rhizobacter, Rhodanobacter, Arenimonas, Amaricoccus, Phyllobacterium, Rhizobium, Pseudomonas, Phaselicystis,* and *Haliangium*. Furthermore, the aforementioned genera had significant higher counts under eCO_2_ conditions according to DESeq2 and Aldex2 results. From other experiments, it was also reported that under eCO_2_ conditions the rhizosphere soil microbial communities had changed [78]. Increased atmospheric CO_2_ concentrations could also change the competitive ability of *Rhizobium leguminosarum* bv. *trifolii*, probably due to changes in root exudates [79]. In salt marsh systems containing the halophyte *Suaeda japonica*, it was reported that gene abundances and microbial community structures were both affected by eCO_2_, and rhizospheric microorganisms responded to eCO_2_ more strongly than those inhabiting the bulk soil [80]. Song et al. [81] described that community composition of soil microbiota associated with *Phytolacca americana* and *Amaranthus cruentus* roots were significantly affected by eCO_2_, and numbers of bacteria and fungi, as well as microbial C and N in the rhizosphere soils of both species, were higher at eCO_2_.

Greater carbon input due to eCO_2_ also explains the increase of 35% in soil CO_2_ fluxes and the 20% augmentation in 16S rRNA copy numbers from active bacterial biomass observed in rhizosphere soil under eCO_2_ in comparison to aCO_2_, which corresponded to an increased soil biological activity in Gi-FACE. Cheng et al. [82] described that eCO_2_ affected soil microbial respiration, producing an augmentation of microbial biomass and activities. Similarly, King et al. [83] showed that eCO_2_ increased soil respiration at four forest FACE experiments. Blagodatskaya et al. [84] demonstrated that augmented available organic C released by roots at eCO_2_ altered the ecological strategy of the soil microbial community, occurring a shift to a higher contribution of fast-growing species. The increased biological activity in eCO_2_ rhizosphere soil is supported by the predicted functional metagenome obtained with PICRUSt2, which shows significant increases in several enzymes involved in cellular components biosynthesis such as peptidoglycan, lipopolysaccharide, amino acids, bacterial motility proteins, and lipids synthesis (S5). These results differ from those obtained by Pujol Pereira et al. [23], who reported that on soybean [*Glycine max* (L.) Merr.], eCO_2_ decreased 16S rRNA gene abundance in rhizosphere soil by 31%. Also, Marhan et al. [21] described that abundances of total 16S rRNA were not influenced by CO_2_ but by sampling date and depth. Likewise, Brenzinger et al. [27] and Bei et al. [28] reported that at the Gi-FACE no differences between aCO_2_ and eCO_2_ rings were found regarding the 16S rRNA gene. However, Bei et al. [28] described that in summer of 2015 under eCO_2_ conditions the functional metagenome of rhizosphere soil presented an increase on amino acids and carbohydrates metabolisms, membrane transporters, and quorum sensing proteins: similar to our study’s PICRUSt2 results (S5).

In addition, several genera involved in the degradation of carbon (C) compounds were stimulated under eCO_2_ conditions; among these are *Pseudomonas* and *Bacillus* (Fig. 4, S2) that have been previously reported to degrade lignocellulose materials. *Pseudomonas boreopolis* produces a cellulase-free xylanase with a high activity of hemicellulose degradation [85]. Maki et al. [86] reported that *Bacillus* strain (55S5) and a *Pseudomonas* strain (AS1) displayed high potential for lignocellulose decomposition due to a variety of cellulase and xylanase activities. Trujillo-Cabrera et al. [87] described the isolation of cellulolytic bacteria from high humus content soils, as *Bacillus thuringiensis* and *Pseudomonas gessardii*. The augmentation of these taxa would agree with the predicted functional metagenome, which indicated an increment of several enzymes involved in lignocellulose materials degradation, as Chitinase (EC:3.2.1.14), Endo-1,3(4)-beta-glucanase (EC:3.2.1.6), Endo-1,4-beta-xylanase (EC:3.2.1.8), and Cellulase (EC:3.2.1.4) (S5). Similar results have been described by He et al. [17, 19], who reported that soils of a soybean agro-ecosystem and a glacial outwash sandplain showed increased abundance of encoding genes for enzymes involved in labile C degradation such as amylase, glucoamylase, pullulanase, fungal arabinofuranosidase, xylanase, endoglucanase, acetylglucosaminidase, and exochitinase. Likewise, Xiong et al. [20] described that alpha-amylase, cellobiase, endoglucanase, vanillin dehydrogenase, endochitinase, and phenoloxidase encoding genes were stimulated under eCO_2_ in soybean and maize fields. The above-mentioned increase of C compounds degradation could occur as a response of greater C availability due to an increase of root exudates under eCO_2_ conditions.

Moreover, these changes in the C influx could induce a reduction of available N in the soil ecosystem [24], which alters the N cycle and induces significant changes in soil biogeochemical characteristics in the rhizosphere, such as NO_3_^−^, available K^+^, soil microbial biomass carbon (SMBC), and available PO_4_^2−^ [78]. The aforementioned process could explain the higher carbon/nitrogen ratio found in our study in eCO_2_ rings in comparison with ambient ones and might also explain why some genera involved in different processes of the nitrogen cycle were stimulated under eCO_2_ conditions. Genera belonging to families Rhizobiaceae and Xanthobacteraceae as *Rhizobium*, *Mesorhizobium*, and *Phyllobacterium* have been extensively reported as nitrogen-fixing bacteria [88–90], and in our study presented Log_2_ fold increases ranging from 6.78 to 8.04. Also, PICRUSt2 results indicate that functional orthologs of the enzyme nitrogenase (EC:1.18.6.1) were significantly augmented in eCO_2_ rhizosphere soil (S5). This increase in the abundance of nitrogen-fixing bacteria could have occurred as response to N deficiency, which eventually became a limiting factor for biomass production under eCO_2_. Similar results were reported by Li et al. [91], who described a 24% increment of ^15^ N in mine tailing soils under eCO_2_ and a dominance of uncultured nitrogen-fixing bacteria.

Aldex2 correlation results demonstrated a significant negative correlation between NH_4_^+^ content and *Nitrospira* genus under eCO_2_ conditions. Although NH_4_^+^ values were not significantly different between aCO_2_ and eCO_2_, NH_4_^+^ content was on average 10% higher in aCO_2_ soils, which suggest that nitrification processes could have been affected due to elevated environmental CO_2_. Alterations in nitrification process in the Gi-FACE have been already described by Müller et al. [72], who reported that eCO_2_ reduced NH_4_^+^ oxidation to NO_3_^−^ by 25%.

Furthermore, several denitrifying genera as *Streptomyces, Rhodanobacter, Pseudomonas, Burkholderia*, and *Bacillus* were significantly stimulated in eCO_2_ rhizospheric soil with Log_2_ fold changes between 5.37 and 8.065 [92–96]. Additionally, predicted functional metagenome indicate that several orthologs involved in the denitrification process, as nitric-oxide synthase (NAD(P)H) (EC:1.14.13.165), nitrate reductase (EC:1.7.99.4), nitrite reductase (NO-forming) (EC:1.7.2.1), nitrite reductase (cytochrome; ammonia-forming) (EC:1.7.2.2), periplasmic nitrate reductase NapA (EC:1.7.99.-), and nitric oxide reductase NorD protein, have significantly greater abundance under eCO_2_ conditions (S5).

**Fig. 6:**
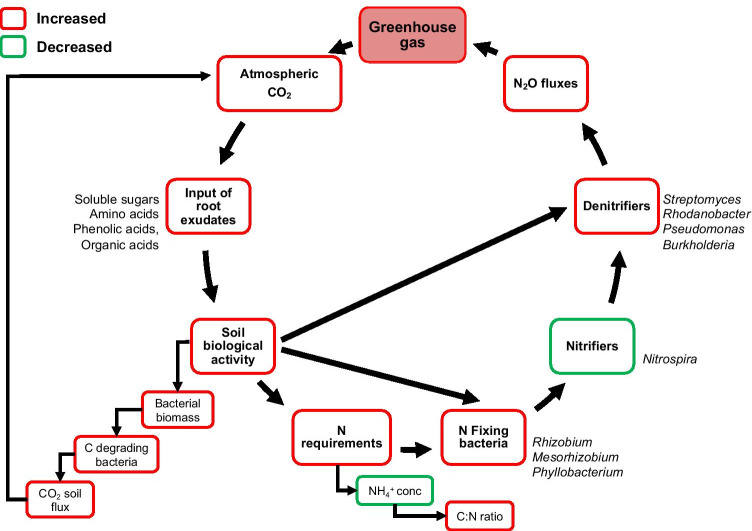
Model diagram of the effect of elevated CO_2_ on the rhizosphere microbiome of C and N cycles bacterial taxa of the Giessen FACE experiment

In summary, our results demonstrate that in the Gi-FACE, the rhizosphere soil microbiome was significantly affected due to the influence of increased CO_2_ concentrations alongside other environmental factors. The increment of carbon input due to eCO_2_ possibly augmented labile carbon degradation in rhizosphere soil reflected by the increment of bacteria biomass and CO_2_ soil emissions. The aforementioned processes could cause a nitrogen constraint, observed in the increment of the C:N ratio, and decreased of NH_4_^+^, which likely triggered a shift in the rhizosphere soil microbiome with an increment of nitrogen fixing and denitrifying taxa. The observed increase of denitrifier genera might explain the increased N_2_O fluxes under eCO_2_ conditions, previously described in the Giessen FACE [27, 97, 98]. Similarly, our data support the results described by Moser et al. [98], who reported that under eCO_2_ conditions, N_2_O emissions were 1.79-fold higher and that the linear interpolations showed a 2.09-fold increase in N_2_O emissions mostly because of the oxidation of organic N and incomplete reduction of NO_2_^−^, emitting N_2_O instead of N_2_ (Fig. 6).

Our findings suggest that alterations in carbon cycle affects nitrogen cycle dynamics in grassland soils, due to changes on the microorganisms involved on the different processes of these cycles. Nonetheless, further analyses would be necessary to assess the Gi-FACE microbiome metatranscriptome of carbon and nitrogen cycles, how they are affected by eCO_2_, and how this effect depends on ambient temperature regimes like summer heat waves.

## Supplementary Information

Below is the link to the electronic supplementary material.

Supplementary file1 (DOCX 668 KB)

Supplementary file2 (XLSX 101 KB)

Supplementary file3 (XLSX 73 KB)

Supplementary file4 (DOCX 891 KB)

Supplementary file5 (XLSX 649 KB)

## Data Availability

The authors declare that the data supporting the findings of this study are available within the article and its Supplementary Information. cDNA sequence data are available in the GenBank database under the accession number PRJNA656997.
