# Supplementary material for: Elevated Atmospheric CO2 Modifies Mostly the Metabolic Active Rhizosphere Soil Microbiome in the Giessen FACE Experiment
Source: Microb Ecol. 2021 Jun 19;83(3):619–34. doi: 10.1007/s00248-021-01791-y (PMC8979872; doi:10.1007/s00248-021-01791-y)
Supplement: Supplementary file 1 — Supplementary file1 (DOCX 668 KB) [file 248_2021_1791_MOESM1_ESM.docx]

**Supplementary material 1**

**Elevated atmospheric CO_2_ modifies mostly the active rhizosphere soil microbiome in the Giessen FACE experiment**

David Rosado-Porto, Stefan Ratering, Massimiliano Cardinale, Corinna Maisinger, Gerald Moser, Marianna Deppe, Christoph Müller, Sylvia Schnell

**Beta diversity dispersion of soil cores per ring**

For evaluation of the differences between the four replicate soil core samples within the six rings beta diversity dispersion was analysed, after centered log ratio transformation, creation of a distance matrix on an euclidian space and an ordination using Principal Components method.

**
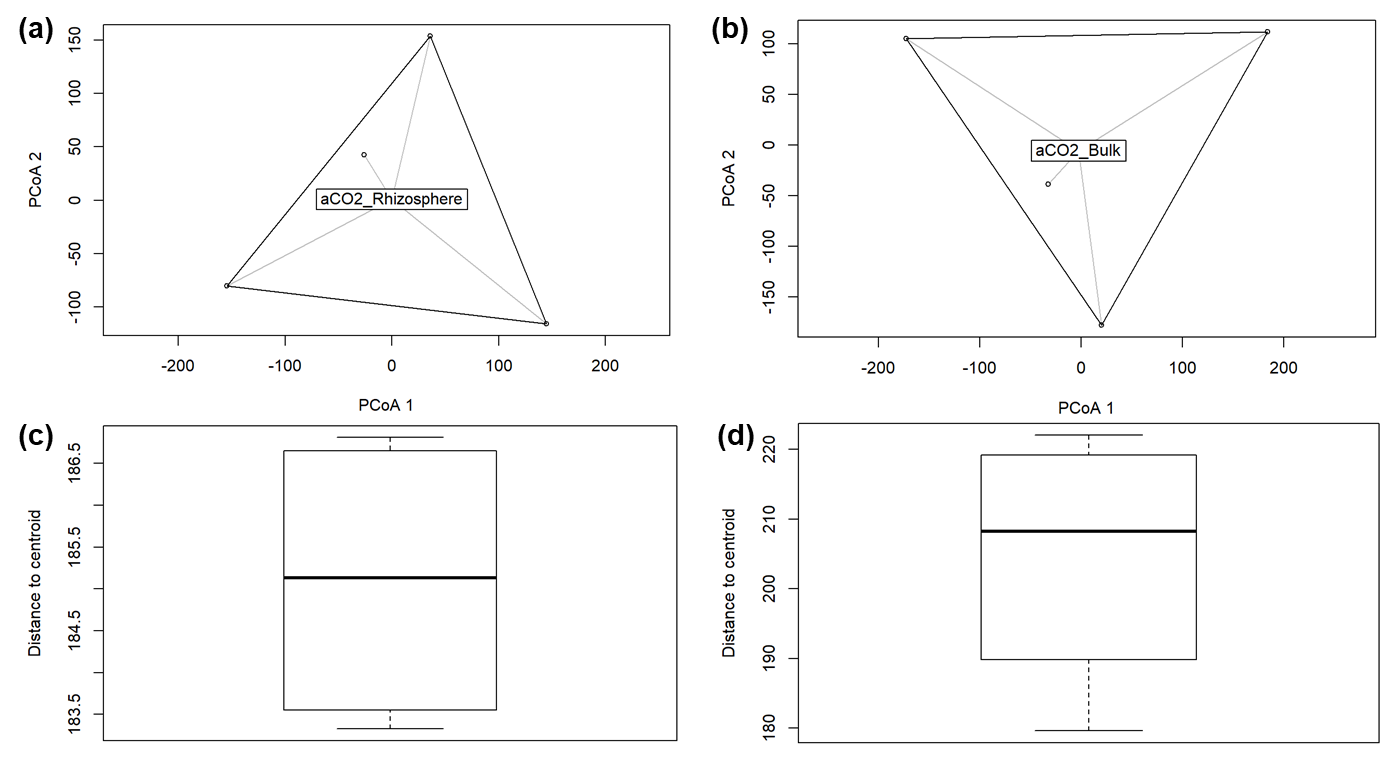
Dispersion of soil cores from ring ambient CO_2_ A1**

**Figure 1. a)** Rhizosphere soil cores distance to the centroid on the Aitchison distance space. **b)** Bulk soil cores distance to the centroid on the Aitchison distance space. **c)** Boxplot of the dispersion of the four rhizosphere soil cores to the centroid. **d)** Boxplot of the dispersion of the four bulk soil cores to the centroid.

| Soil core | Rhizosphere soil | |  | Bulk soil | |
| --- | --- | --- | --- | --- | --- |
|  | PCoA1 | PCoA2 |  | PCoA1 | PCoA2 |
| 1 | -25.80094 | 42.39630 |  | 183.98338 | 111.6005 |
| 2 | 144.69421 | -115.87543 |  | -172.43575 | 105.1300 |
| 3 | -154.55176 | -80.12298 |  | 20.50771 | -177.9165 |
| 4 | 35.65849 | 153.60212 |  | -32.05535 | -38.8139 |
| Centroid | **0.08293701** | **0.7826727** |  | **-2.238718** | **-5.915377** |

**
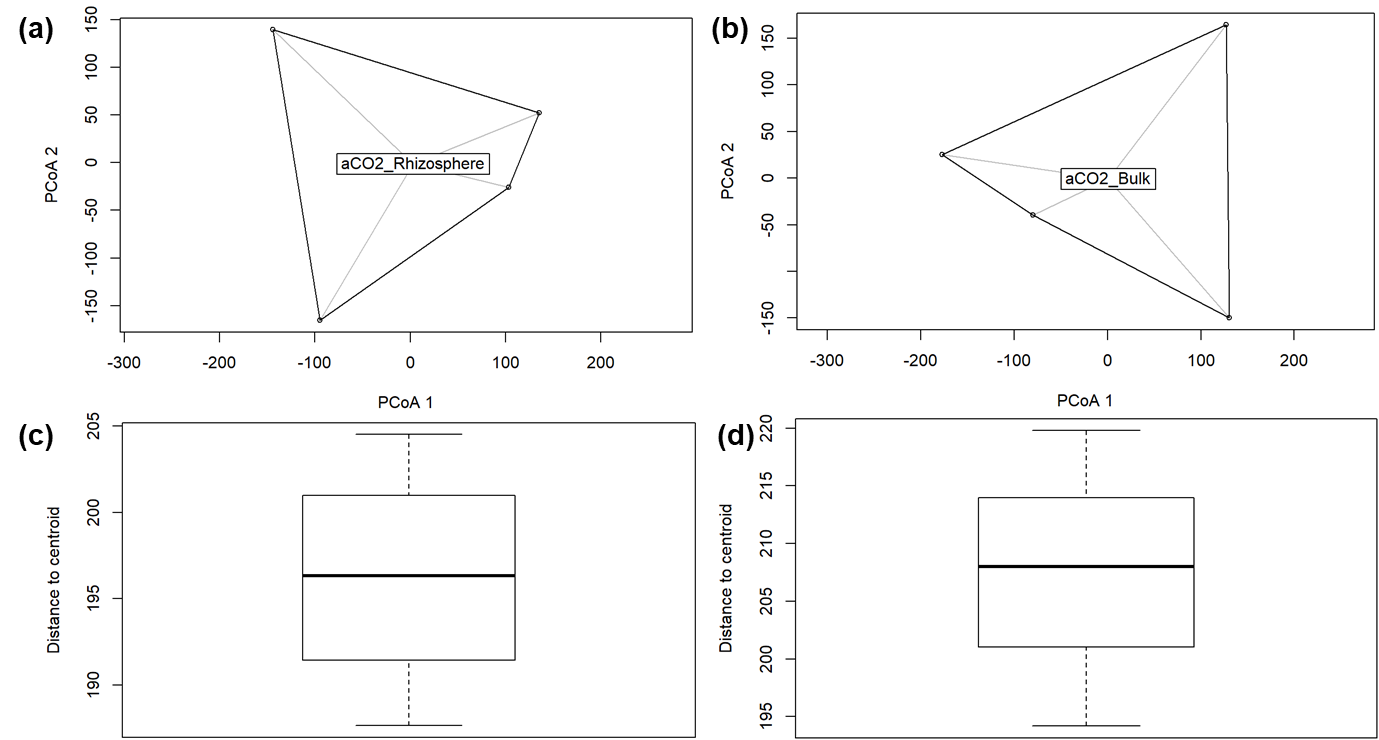
Dispersion of soil cores from ring ambient CO_2_ A2**

**Figure 2. a)** Rhizosphere soil cores distance to the centroid on the Aitchison distance space. **b)** Bulk soil cores distance to the centroid on the Aitchison distance space. **c)** Boxplot of the dispersion of the four rhizosphere soil cores to the centroid. **d)** Boxplot of the dispersion of the four bulk soil cores to the centroid

| Soil core | Rhizosphere soil | |  | Bulk soil | |
| --- | --- | --- | --- | --- | --- |
|  | PCoA1 | PCoA2 |  | PCoA1 | PCoA2 |
| 1 | 135.59410 | 52.11665 |  | 130.48605 | -149.88747 |
| 2 | 103.29415 | -26.04585 |  | 126.89718 | 164.30589 |
| 3 | -94.88099 | -165.50639 |  | -177.36804 | 25.11917 |
| 4 | -144.00725 | 139.43559 |  | -80.01519 | -39.53759 |
| Centroid | **2.953534** | **-1.379265** |  | **0.900758** | **-0.9933014** |

**
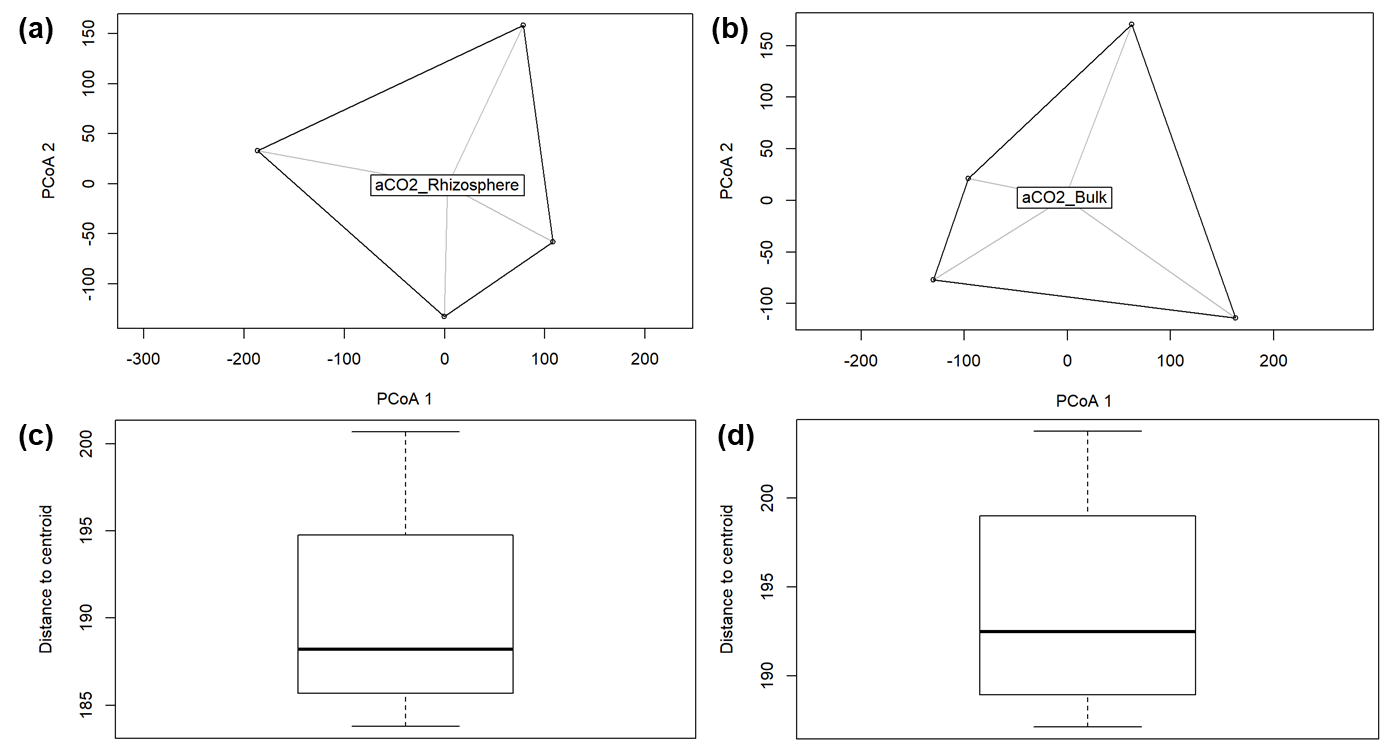
Dispersion of soil cores from ring ambient CO_2_ A3**

**Figure 3. a)** Rhizosphere soil cores distance to the centroid on the Aitchison distance space. **b)** Bulk soil cores distance to the centroid on the Aitchison distance space. **c)** Boxplot of the dispersion of the four rhizosphere soil cores to the centroid. **d)** Boxplot of the dispersion of the four bulk soil cores to the centroid.

| Soil core | Rhizosphere soil | |  | Bulk soil | |
| --- | --- | --- | --- | --- | --- |
|  | PCoA1 | PCoA2 |  | PCoA1 | PCoA2 |
| 1 | 78.745905 | 158.24572 |  | 163.44855 | -114.17100 |
| 2 | 108.168563 | -58.39199 |  | -96.01281 | 20.96616 |
| 3 | -186.607149 | 33.14931 |  | -129.89925 | -77.20935 |
| 4 | -0.307318 | -133.00304 |  | 62.46351 | 170.41419 |
| Centroid | **2.950533** | **-1.509491** |  | **-2.558924** | **2.204324** |

**
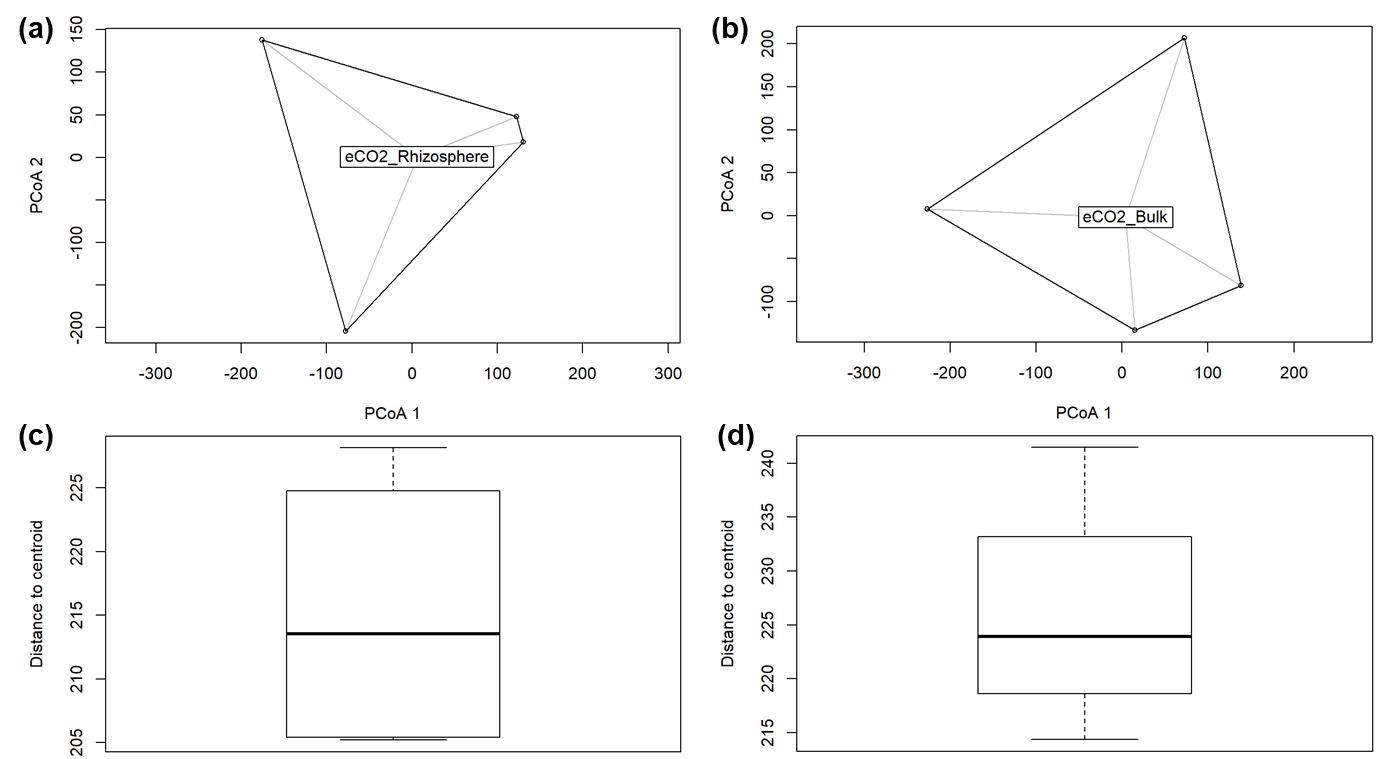
Dispersion of soil cores from ring elevated CO_2_ E1**

**Figure 4. a)** Rhizosphere soil cores distance to the centroid on the Aitchison distance space. **b)** Bulk soil cores distance to the centroid on the Aitchison distance space. **c)** Boxplot of the dispersion of the four rhizosphere soil cores to the centroid. **d)** Boxplot of the dispersion of the four bulk soil cores to the centroid.

| Soil core | Rhizosphere soil | |  | Bulk soil | |
| --- | --- | --- | --- | --- | --- |
|  | PCoA1 | PCoA2 |  | PCoA1 | PCoA2 |
| 1 | 122.64175 | 48.16592 |  | 72.67961 | 206.641682 |
| 2 | 130.62563 | 18.26833 |  | 138.75110 | -81.113733 |
| 3 | -77.77673 | -204.18850 |  | 14.83166 | -133.417941 |
| 4 | -175.49064 | 137.75424 |  | -226.26238 | 7.889992 |
| Centroid | **6.037281** | **0.2841273** |  | **4.394225** | **-1.951842** |

**
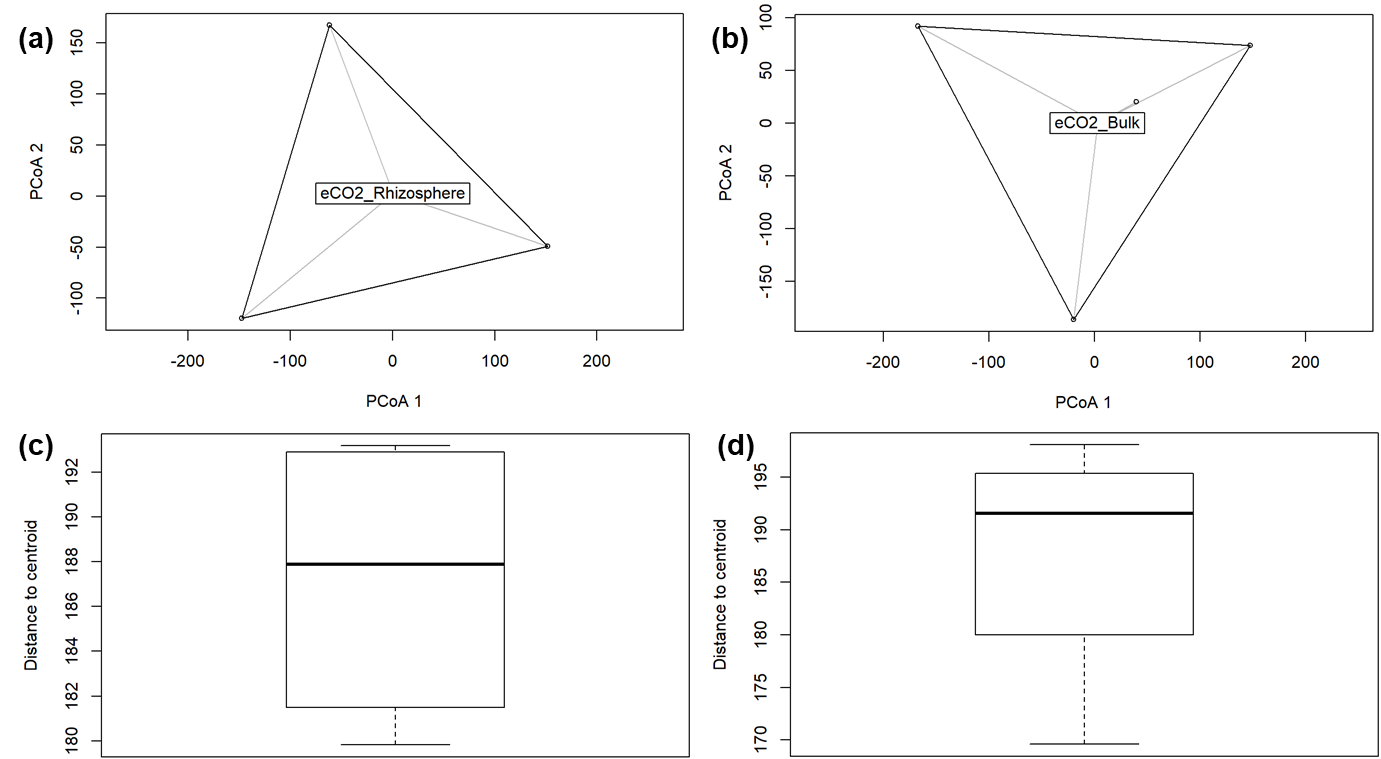
Dispersion of soil cores from ring elevated CO_2_ E2**

**Figure 5. a)** Rhizosphere soil cores distance to the centroid on the Aitchison distance space. **b)** Bulk soil cores distance to the centroid on the Aitchison distance space. **c)** Boxplot of the dispersion of the four rhizosphere soil cores to the centroid. **d)** Boxplot of the dispersion of the four bulk soil cores to the centroid.

| Soil core | Rhizosphere soil | |  | Bulk soil | |
| --- | --- | --- | --- | --- | --- |
|  | PCoA1 | PCoA2 |  | PCoA1 | PCoA2 |
| 1 | 57.78793 | 1.541002 |  | 147.46292 | 73.87424 |
| 2 | 151.76817 | -48.972109 |  | -19.70307 | -186.11782 |
| 3 | -147.68316 | -119.776634 |  | -167.19865 | 91.88826 |
| 4 | -61.87294 | 167.207742 |  | 39.43880 | 20.35531 |
| Centroid | **0.3317109** | **2.198898** |  | **2.836266** | **0.2872584** |

**
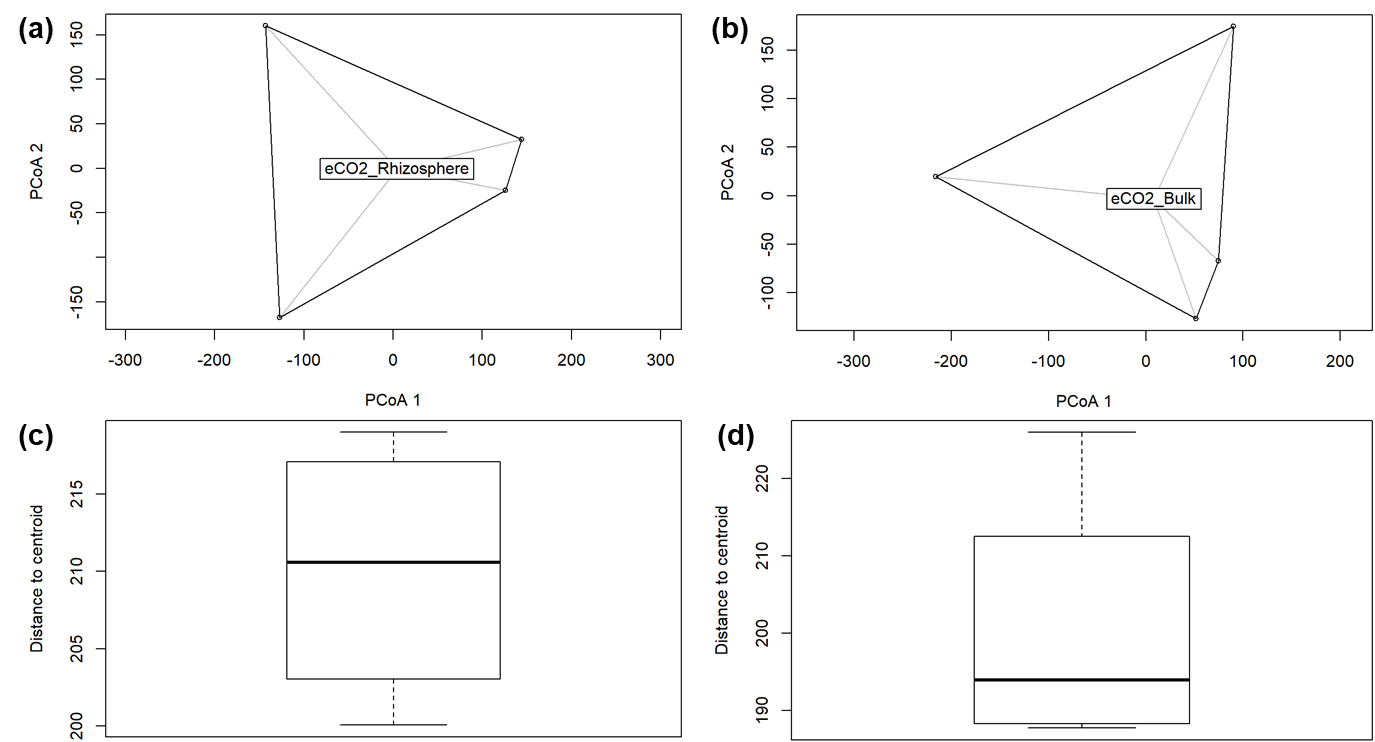
Dispersion of soil cores from ring elevated CO_2_ E3**

**Figure 6. a)** Rhizosphere soil cores distance to the centroid on the Aitchison distance space. **b)** Bulk soil cores distance to the centroid on the Aitchison distance space. **c)** Boxplot of the dispersion of the four rhizosphere soil cores to the centroid. **d)** Boxplot of the dispersion of the four bulk soil cores to the centroid.

| Soil core | Rhizosphere soil | |  | Bulk soil | |
| --- | --- | --- | --- | --- | --- |
|  | PCoA1 | PCoA2 |  | PCoA1 | PCoA2 |
| 1 | 144.0663 | 32.32366 |  | 74.47449 | -66.96166 |
| 2 | 125.9443 | -24.58944 |  | 51.56435 | -126.67887 |
| 3 | -127.0612 | -167.76252 |  | -216.33316 | 19.45550 |
| 4 | -142.9494 | 160.02830 |  | 90.29432 | 174.18503 |
| Centroid | **4.478243** | **-0.7855918** |  | **8.294987** | **-3.311289** |

**Conclusions**

The beta diversity results and its dispersion for each ring show that the cores taken per ring are different enough to be at different positions on the Aitchison space. Furthermore, when analyzing the dispersion and their distance to the centroids, all of them showed a fairly great level of dispersion, which leads us to think that the soil cores although they are very similar among them, also they are sufficiently different to not be considered as technical replicates, but single samples.
