# Supplementary material for: Elevated Atmospheric CO2 Modifies Mostly the Metabolic Active Rhizosphere Soil Microbiome in the Giessen FACE Experiment
Source: Microb Ecol. 2021 Jun 19;83(3):619–34. doi: 10.1007/s00248-021-01791-y (PMC8979872; doi:10.1007/s00248-021-01791-y)
Supplement: Supplementary file 4 — Supplementary file4 (DOCX 891 KB) [file 248_2021_1791_MOESM4_ESM.docx]

**Supplementary material 4**

**Elevated atmospheric CO_2_ modifies mostly the active rhizosphere soil microbiome in the Giessen FACE experiment**

David Rosado-Porto, Stefan Ratering, Massimiliano Cardinale, Corinna Maisinger, Gerald Moser, Marianna Deppe, Christoph Müller, Sylvia Schnell


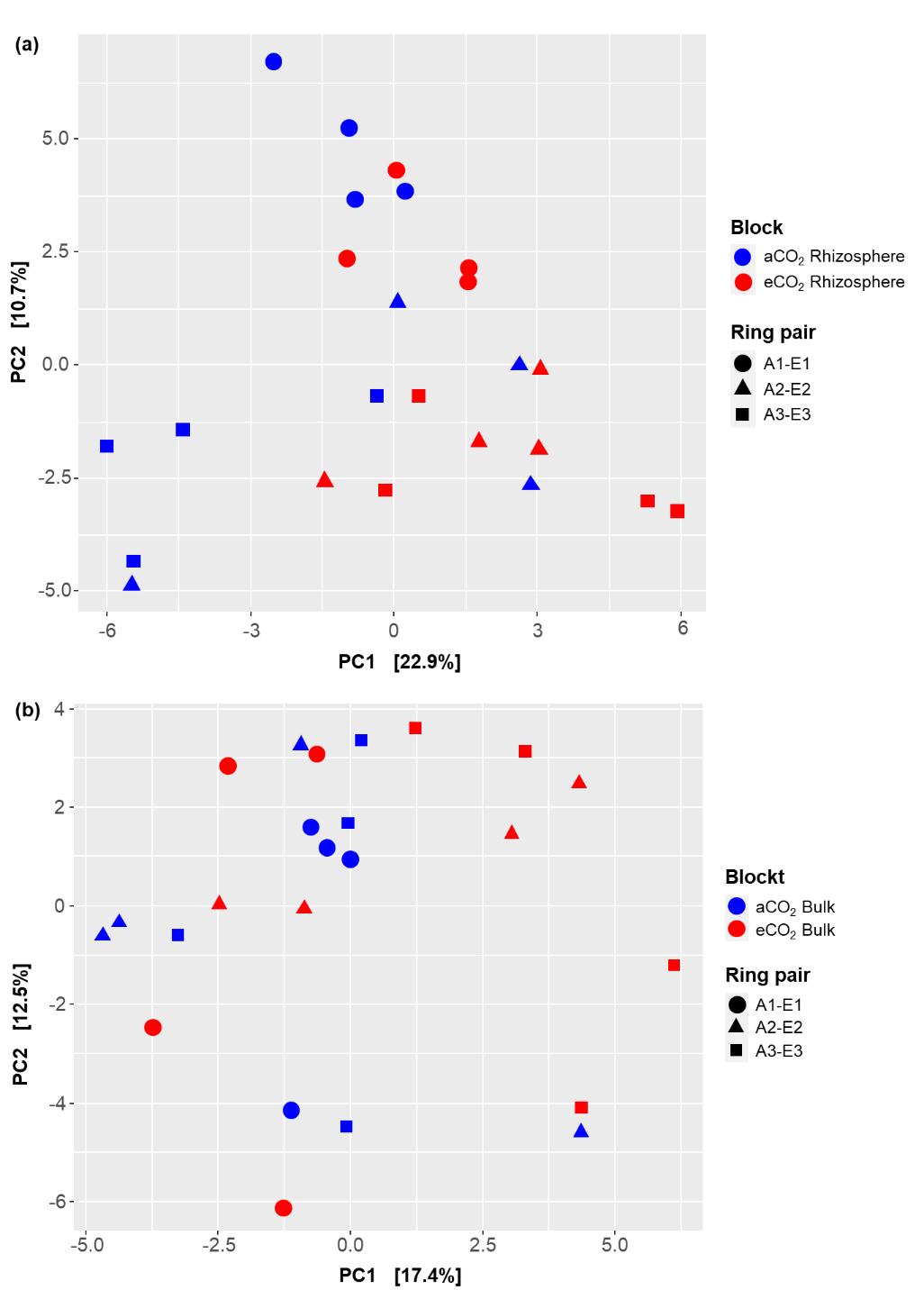


**Figure S4.1**. Principal Components Analysis (PCA) of Enzyme Commission number‘s (EC number) predicted metagenome, calculated based on Aitchison community dissimilarity distance matrix of (a) rhizosphere soils from ambient and elevated CO_2_ rings and (b) bulk soils from ambient and elevated CO_2_ rings. A, ambient CO_2_ rings; E, elevated CO_2_ rings; aCO_2_, ambient CO_2_ conditions; eCO_2_, elevated CO_2_ conditions.

**Tab S4.** Beta diversity p-values of Permutational Multivariate Analysis of Variance Using Distance Matrices of predicted functional metagenome from Gi-FACE’s rhizosphere and bulk soils, calculated based on Aitchison community dissimilarity distance matrix.

| Factor | Rhizosphere soil | | | Bulk soil | | |
| --- | --- | --- | --- | --- | --- | --- |
|  | EC number | KEGG Orthology | MetaCyc  Pathway | EC number | KEGG Orthology | MetaCyc  Pathway |
| Block | 0.005 ** | 0.019 * | 0.022 * | 0.197 | 0.179 | 0.317 |
| Ring | 0.001 *** | 0.002 ** | 0.005 ** | 0.011 * | 0.05 * | 0.342 |

Significance codes: 0.0001 ‘***’ , 0.001 ‘**’ , 0.01 ‘*’ , 0.05 ‘.’.


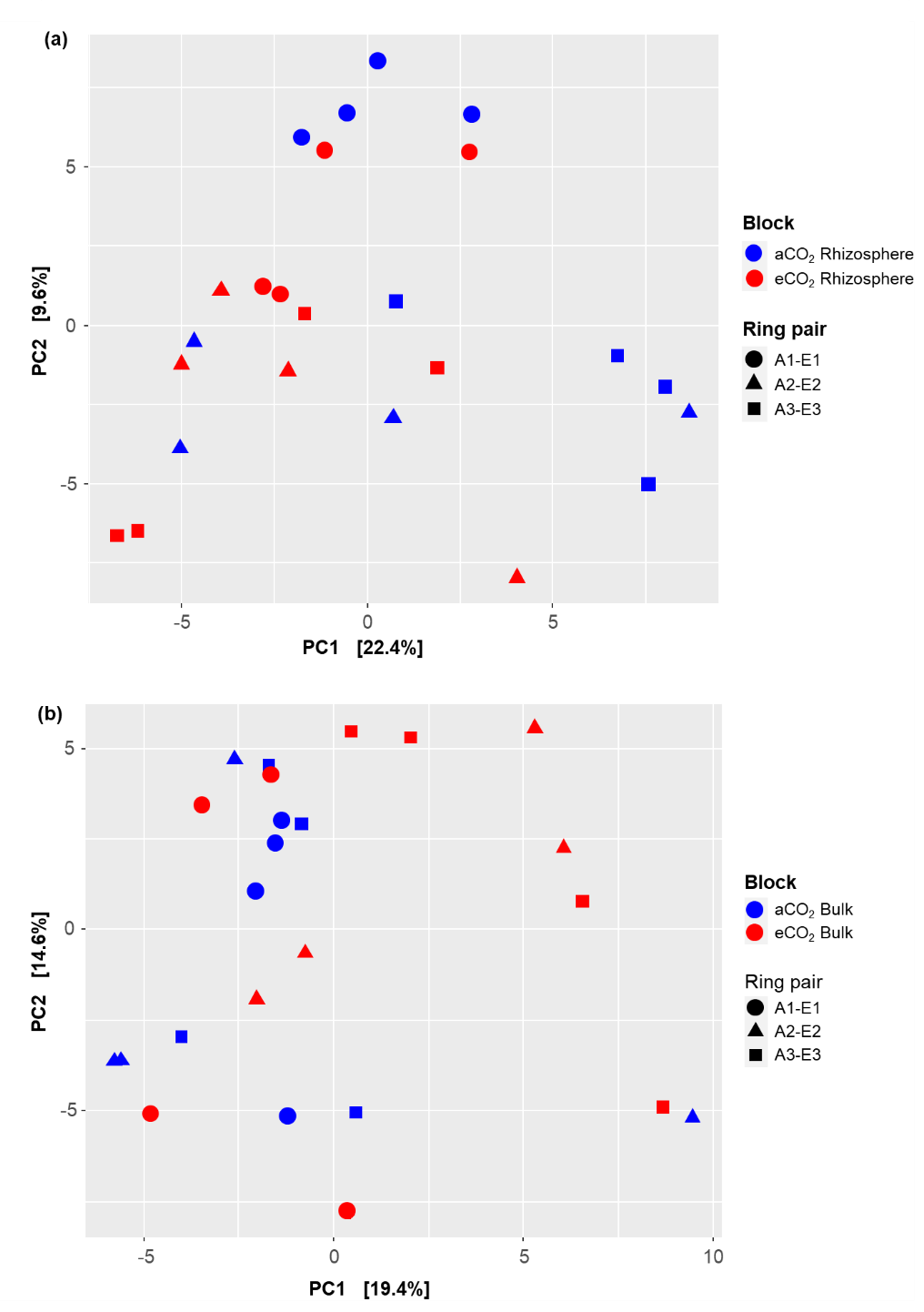


**Figure S4.2.** Principal Components Analysis (PCA) of KEGG Orthology (KO) for molecular functions predicted metagenome, calculated based on Aitchison community dissimilarity distance matrix of (a) rhizosphere soils from ambient and elevated CO_2_ rings and (b) bulk soils from ambient and elevated CO_2_ rings. A, ambient CO_2_ rings; E, elevated CO_2_ rings; aCO_2_, ambient CO_2_ conditions; eCO_2_, elevated CO_2_ conditions.


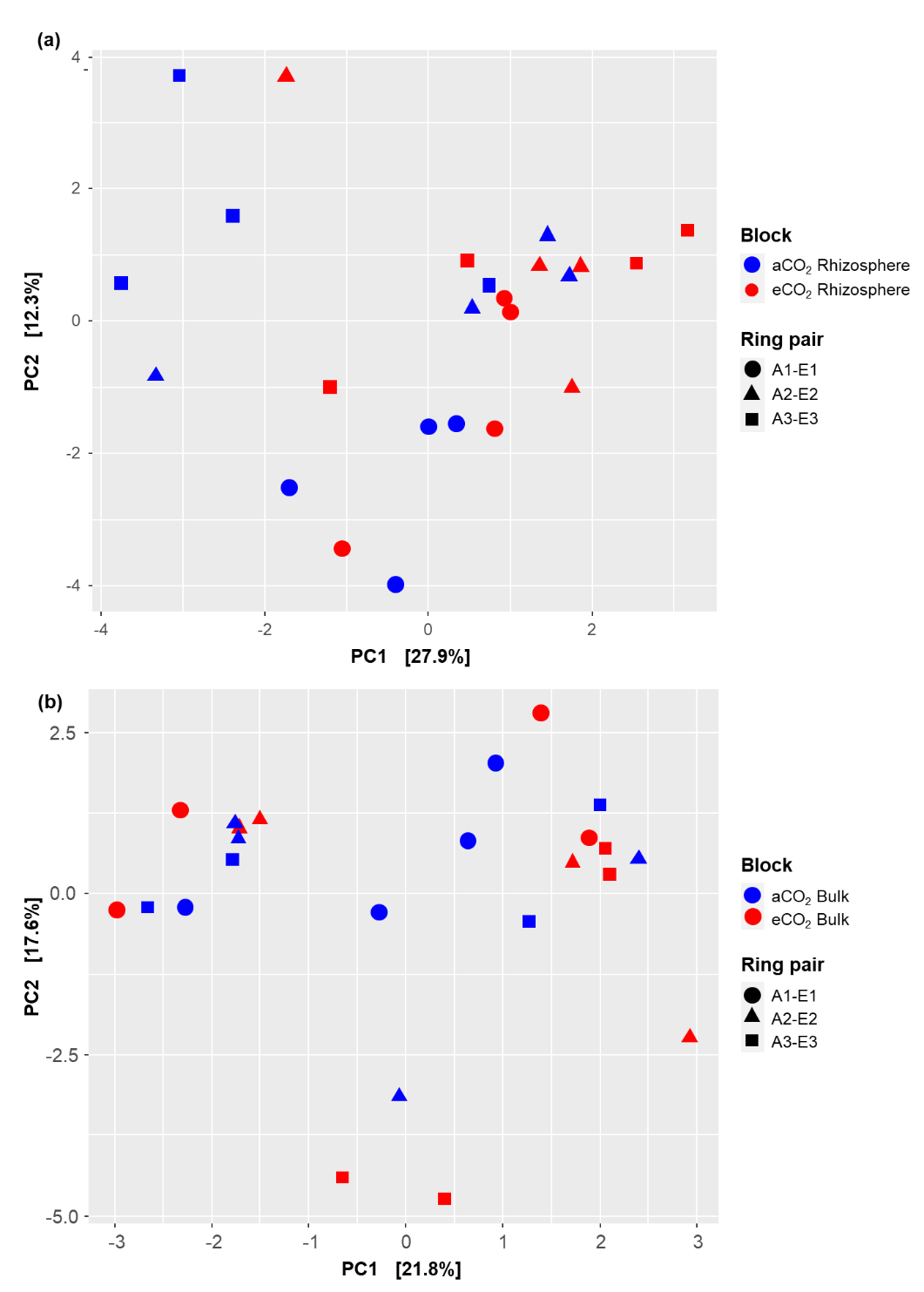


**Figure S4.3.** Principal Components Analysis (PCA) of MetaCyc Metabolic Pathways of the predicted metagenome, calculated based on Aitchison community dissimilarity distance matrix of (a) rhizosphere soils from ambient and elevated CO_2_ rings and (b) bulk soils from ambient and elevated CO_2_ rings. A, ambient CO_2_ rings; E, elevated CO_2_ rings; aCO_2_, ambient CO_2_ conditions; eCO_2_, elevated CO_2_ conditions.


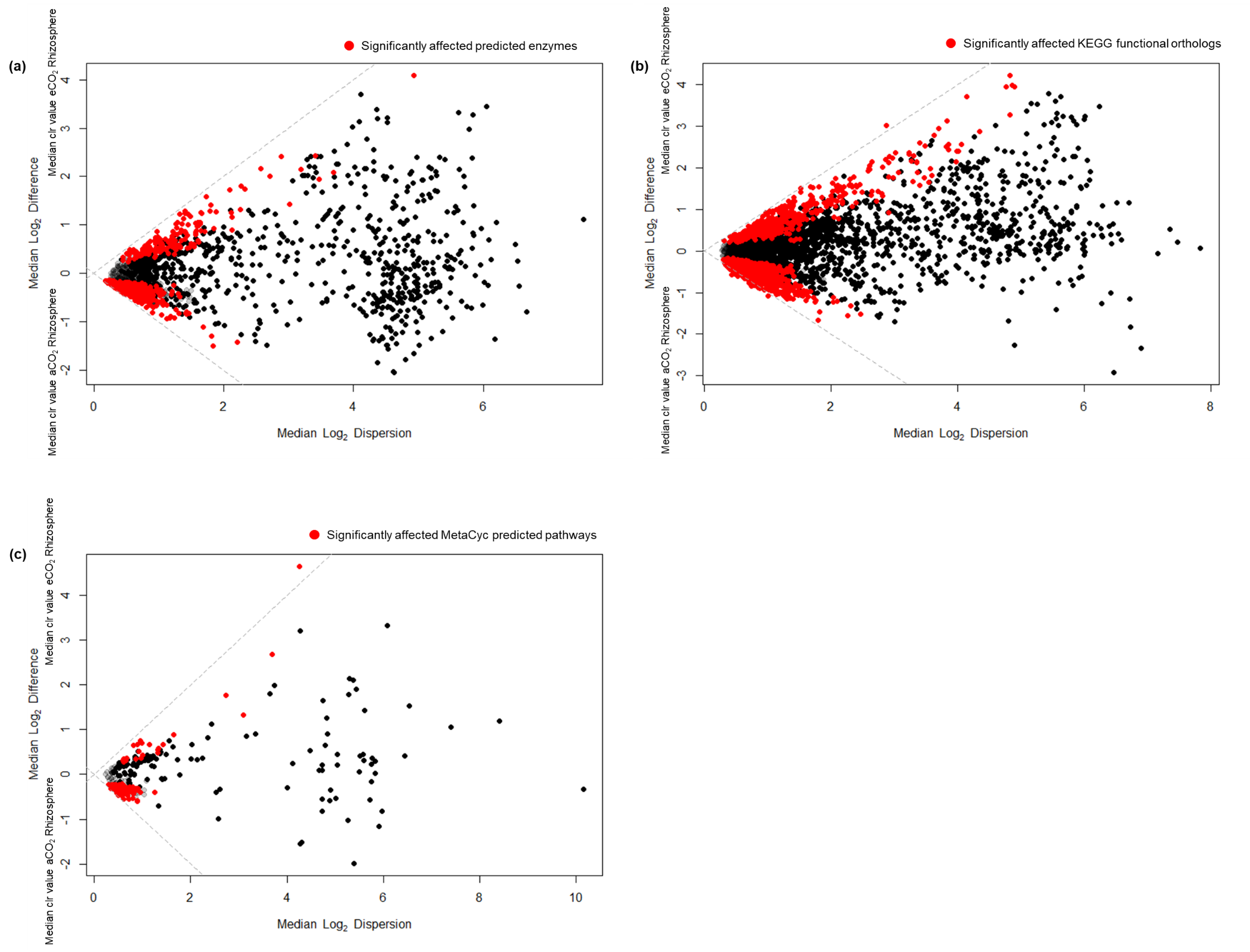


**Figure S4.4.** Differential abundances of predicted metagenome of rhizosphere soil under elevated and ambient CO_2_ of (a) Enzyme Commission number‘s (EC number); (b) KEGG Orthology (KO) for molecular functions; (c) MetaCyc pathways with Aldex2 using centered log ratio (clr) transformation and the geometric mean abundance of all features; red points indicate significantly different features after Welch's t-test and Benjamini-Hochberg correction with an alpha threshold <0.1.
